# Relationship between weight and linear dimensions of Bluefin tuna (*Thunnus thynnus*) following fattening on western Mediterranean farms

**DOI:** 10.1371/journal.pone.0200406

**Published:** 2018-07-10

**Authors:** Vicente Puig-Pons, Vicente Domingo Estruch, Víctor Espinosa, Fernando de la Gándara, Begonya Melich, José Luis Cort

**Affiliations:** 1 Institut d’Investigació per a la Gestió Integrada de Zones Costaneres, Universitat Politècnica de València, Grau de Gandia (Valencia), Spain; 2 Instituto Español de Oceanografía, Centro Oceanográfico de Murcia. Planta de Cultivos Marinos, Puerto de Mazarrón (Murcia), Spain; 3 Grup Balfegó, L’Ametlla de Mar (Tarragona), Spain; 4 Fisheries Area, Instituto Español de Oceanografía, Centro Oceanográfico de Santander, Santander (Cantabria), Spain; National Oceanic and Atmospheric Administration, UNITED STATES

## Abstract

This study presents various models based on formulae relating weight and dimensions (length, height and width) of Bluefin tuna, *Thunnus thynnus* (L.), fattened in captivity. The main aim of establishing these expressions is to design tools for indirectly predicting the weight of a Bluefin tuna from measurements of one or more dimensions obtained using non-invasive methods such as stereoscopic cameras. Measurements of maximum length, height and width following slaughter were taken of fish fattened in captivity (n = 2078). Different relationships drawn from the dimensions of the tuna against their weight are fitted with part of the data collection and later checked against a reserved sample set. The resulting formulae are compared with the formulae most commonly used in the case of wild tuna. The results of this study confirm that, for tuna fattened in cages, the availability of more than one dimension to estimate weight improves the predictive power of the model and reduces error in the estimate.

## Introduction

Atlantic Bluefin Tuna—ABFT- is a highly migratory species. The International Commission for the Conservation of Atlantic Tunas (ICCAT) is responsible for managing ABFT, and as part of the management of this species, ICCAT split the stock management units into two parts, West stock and East stock (which includes the Mediterranean). The boundary between the two stocks is defined as the 45° W meridian, though in reality high rates of mixing have been found, which vary from one year to another [1,2]. Tuna farming based on adult capture in breeding areas has recently been developed. In the Mediterranean Sea, ABFT is fished from May to July by a purse-seining fleet. After capture, tuna are transferred into towing cages (usually circular with a 50 m diameter) and transported to permanent cages near the coast. Transport may take several weeks because to decrease stress and mortalities tuna are transported at very low speed (1 knot) [3,4]. Once the towing cage arrives at the fish farm, tuna are transferred to the fattening cages and an effort is made to assess the biomass. As a rule, aquaculture farms carry out manual sampling to obtain mean weight data, however, in the case of ABFT these samplings are very costly and require very difficult manoeuvers that greatly stress the fish and lead to unwanted deaths. For this reason, samplings are usually made with systems using stereoscopic cameras, which provide recordings of the fish from the side in a non-invasive way [5,6,7,8]. These recordings permit fish lengths to be evaluated by measuring them from each pair of stereoscopic images. From this information, mathematical relationships are applied to estimate weight. ICCAT has established this procedure as an obligatory measurement for catches taken by purse seiners destined for fattening in cages, with implementation to take place during the process of transferring tuna to the fattening cages [9]. In addition to length, some of these stereoscopic systems permit maximum fish height to be used in the estimation of weight [10].

For farming purposes, ABFT are housed in fattening cages typically located in close proximity to the shore. A target density in these cages is ≤ 6 kg fish/m3 [4,11]. Fattening period in the Mediterranean sea runs from July to February, during the fattening period ABFT are fed a variety of defrosted small pelagic fish (depending on availability and price): sardinelle (Sardinella aurita), pilchard (Sardina pilchardus), herring (Clupea harengus), mackerel (Scomber scombrus), horse mackerel (Trachurus sp.), chub mackerel (Scomber japonicus), bogue (Boops boops), and some cephalopods [12]. Usually fish are offered 1–3 daily meals but it may increase further (around six times per day) depending on water temperature, tuna size and fish feeding responses. Due to the farming procedure, ABFT morphometrics are modified. For this reason, the use of biometric relationships of wild fish to estimate caged fish weights, offer a mere approximation at best. Moreover, the presence of large and small tuna in the caged fish causes inaccurate weight estimations [4,13,14].

Puig et al. (2012) [15] proposed the measurement of tuna size from the ventral perspective with a pair of stereoscopic cameras, studying the correlation of fish length with measurements of its acoustical target strength. In that experiment, a synchronized system was set up involving a scientific echosounder and a pair of stereoscopic cameras placed in the bottom of the cage and directed upwards to collect images from underneath, unlike the usual configuration in which lateral images are taken. This configuration provides greater quality images when visibility is poor, and has recently been used to obtain automatically accurate length measurements using stereoscopic cameras recording ventral perspective of tuna [16]. In that study, a deformable model of the fish ventral silhouette was developed to measure length and maximum width in a pair of images. Variation of maximum width was proposed to monitor fattening processes. Having new biometric relationships involving these dimensions may facilitate a deeper understanding of the fattening process of captured tuna in farms, but also a better definition of the physiological condition of wild tuna from biometrics. The validity of a specific equation relating weight and length for ABFT, depending on physiological condition associated to different factors (geographical location, season, spawning period, etc) has been a matter of controversy [17,18]. The idea of considering other dimensions in addition to length is not new, since conversion equations including length and height have been introduced before [10]. Nevertheless it is the first time to our knowledge that three dimensions have been considered with the objective of reducing estimate error. This study examines the relationships between weight and different biometric measurements: length, maximum height and maximum width of tuna using statistically significant fits with clear predictive value. It is hoped the results will complement the knowledge from existing studies centered on relating ABFT weight and length, which have not previously considered any other dimension of fish. The results could be of great relevance in the definition of future catch control procedures in different fisheries and species.

## Material and methods

Biometric data from tuna fattening were provided by Grup Balfegó. Data were taken after tuna harvest at a Grup Balfegó farm off the coast of l´Ametlla de Mar in Tarragona (Spain, 4°052’11.7” N and 0°48’15.2” E). These biometrics were obtained in 2012 and 2013 and provided information from n = 2078 tuna. The information included data on length, gross weight, height and sex. Sizes vary between 126 and 273 cm in length with average length of 207.17±17.61 cm (and a median of 207 cm). Gross weight oscillates between 46.29 and 457.14 kg with a mean value of 197.44± 54.76 Kg (191.65 kg median). Height measurements were taken for all samples obtaining a range of values between 33 and 79 cm (56.5±5.72 cm average value). Furthermore, for 1067 of these fish the maximum width was known, with maximum width ranging between 25 and 62 cm and an average value of 43.27±6.74 cm.

A maximum length (Lmax) of ABFT of 331.2 cm (Lmax = 319.93±11.3 cm), established by the most recently published studies, was applied as indicated in the paper by Cort et al. (2013) [19]. Thus, the data were filtered to eliminate all fishes whose lengths exceeded the extremal value, with the aim of obtaining a representative and realistic population sample and preventing undesirable effects on the end results due to outliers.

Using the filtered data, least-squares fitting was performed taking into account diverse mathematical models whose expressions consider different fish dimensions to be predictor variables. Thus, we obtained relationships of the weight to one or more dimensions considering height, length and maximum width. With the aim of finding and verifying relationships between tuna linear dimensions, other expressions relating height to length and maximum width to length were also studied. The equations of the obtained biometric models are shown in Tables 1 and 2.

**Table 1:** Model identifier, equation for the proposed models, and equation for the linearised models and parameters to calculate.

Model identifier	Equation	Linearised equation	Parameters
**M1**	$W=a\cdot L^{2}\cdot H$	—	*a*
**M2**	$W=a\cdot {\left(L+H\right)}^{b}$	$\log_{e}(W)=\alpha +b\cdot \log_{e}(L+H)$	*a*,*b*
**M3**	$W=a\cdot L^{b}\cdot A$	$\log_{e}(W)-\log_{e}(A)=\alpha +b\cdot \log_{e}(L)$	*a*,*b*
**M4**	$W=a\cdot L\cdot A^{b}$	$\log_{e}(W)-\log_{e}(L)=\alpha +b\cdot \log_{e}(A)$	*a*,*b*
**M5**	$W=a\cdot {\left(L+A\right)}^{b}$	$\log_{e}(W)=\alpha +b\cdot \log_{e}(L+A)$	*a*,*b*
**M6**	$W=a\cdot L^{2.06}\cdot A$	—	*a*
**M7**	W=a⋅L⋅H⋅A	—	*a*
**M8**	$W=a\cdot {\left(L\cdot H\cdot A\right)}^{b}$	$\log_{e}(W)=\alpha +b\cdot \log_{e}(L\cdot H\cdot A)$	*a*,*b*
**M9**	$W=a\cdot {\left(L+H+A\right)}^{b}$	$\log_{e}(W)=\alpha +b\cdot \log_{e}(L+H+A)$	*a*,*b*
**M10**	$W=a\cdot L\cdot H^{b}\cdot A^{c}$	$\log_{e}(W)-\log_{e}(L)=\alpha +b\cdot \log_{e}(H)+c\cdot \log_{e}(A)$	*a*,*b*,*c*
**M11**	$W=a\cdot L^{b}\cdot H^{c}\cdot A^{d}$	$\log_{e}(W)=\alpha +b\cdot \log_{e}(L)+c\cdot \log_{e}(H)+d\cdot \log_{e}(A)$	*a*,*b*,*c*,*d*
**M12**	$W=a\cdot L^{b}$	$\log_{e}(W)=\alpha +b\cdot \log_{e}(L)$	*a*,*b*
**M13**	$W=a\cdot A^{3}$	—	*a*

In all cases α = log_e_(*a*)

**Table 2:** Models proposed for the calculation of height from length and width, and the relationship between length and maximum width. The linearised equation is presented for making the linear fit using the least squares method.

Identifier of models	Equation	Linearised equation	Parameters
**M14**	$H={\left(L\cdot A\right)}^{b}$	$\log_{e}(H)=b\cdot \log_{e}(L\cdot A)$	b
**M15**	A=a⋅L	—	a

Table 1 shows models (M1-M13) where the independent variable is weight (W). Models M1 and M2 establish relationships between weight and length (L) and height (H) of the fishes. Models M3, M4, M5 and M6 relate weight with L and maximum width (A). Models M7, M8, M9, M10, and M11 relate weight to the three dimensions of the fishes: L, H A. Finally, models M12 and M13 relate weight with L and A respectively, as usual. Models M6, M7 and M13 are directly linear as a function of the transformations of the predictive variables.

Table 2 shows two models considered to establish relationships between tuna dimensions, height depending on length and maximum width (M14) and maximum width depending on length (M15). To explore the validity of all these models, fits were tested using the data supplied by Grup Balfegó.

To validate the models obtained from the fits, 500 randomly chosen individuals were reserved at the start of the study. The fits were made using the data corresponding to the remaining individuals. The fit to the linearised models was made with the help of Statraphics CENTURION XVI software [20]

All the models considered can be linearised and to avoid computing problems the fits were made using the models once linearised. To determine whether each of the models considered was statistically significant, the F-statistic was calculated, which makes it possible to test the hypothesis that the coefficient of determination, R^2^, is not equal to zero, and therefore the model provides a statistically significant explanation (p-value<0.05) for the relationship between variables. The non-linear degree-of-freedom adjusted (R^2^ (df)) allows evaluation of the variability of the dependent variable explained by the model.

After fitting each model, the degree-of-freedom adjusted coefficient of determination and the F-statistic value(which enables the calculation of p-value) were computed. These indicators were calculated on the linearised models, and are suitable for evaluating the validity of each model separately, but they cannot determine the most suitable model because the linearisation provides non-comparable expressions.

When validating the models, the one proposed in [21] was also considered although this model is used to estimate weight from length in wild fish.

Weight was estimated using the different expressions, and the validity of the models was evaluated by analysing the difference between the real weight value and the weight value estimated by each of the models. Goodness of fit was analyzed with the non-linear degree-of-freedom adjusted coefficient of determination R^2^ (df):

$$R^{2}=1-\frac{\sum_{i}{\left(pr_{i}-pm_{i}\right)}^{2}}{\sum_{i}{\left(pr_{i}-\bar{pr}\right)}^{2}}$$ (1)

$$R^{2}\left(d.f.\right)=R^{2}-\left(1-R^{2}\right)\frac{p-1}{n-p}$$ (2)

where pr is the real weight, pm is the estimated weight, pr is real mean weight, p is the number of explanatory variables and n is the sample size.

Mean absolute error was also calculated (Eam):

$$Eam=\frac{\sum \left|pr_{i}-pm_{i}\right|}{n}Kg$$ (3)

the standard error of the absolute errors (eEa):

$$e_{Ea}=\frac{standard\;deviation\;of\;the\;absolute\;errors}{\sqrt{n}}$$ (4)

the mean of relative errors (Erm):

$$Erm=\frac{\sum \frac{\left|pr_{i}-pm_{i}\right|}{pm_{i}}}{n}$$ (5)

and the standard error of the relative errors (e_Er_)

$$e_{Er}=\frac{s\tan dard\;deviation\;of\;the\;relative\;errors}{\sqrt{n}}$$ (6)

To evaluate whether the predictions of the above expressions and those used for wild fishes under or overestimate weight, the mean value of the residuals was calculated (resm):

$$resm=\frac{\sum pr-pm}{n}$$ (7)

To establish whether there are statistically significant differences between the predictive power of the different models, ANOVA analysis [22] was performed on mean absolute error, mean relative error and mean of the residuals, considering the model as a factor in the three cases. The multiple range test revealed the models between which statistically significant differences are detected (p-value<0.05).

## Results

The results of the fit for the different models considered, obtained from the data facilitated by Grup Balfegó after harvesting tuna fattened at their installations in 2012 and 2013, are shown in Table 3. The table (Table 3) contains the values of the parameters calculated for each model. The values of R^2^(d.f.) correspond to the linearised models and so they are not mutually comparable. The table also includes the degrees of freedom of each model and the value of F, which is used to calculate the p-value associated with the test to check whether the model is statistically significant (R^2^(d.f.) ≠0). The results indicate that, in all cases the models are statistically significant for a confidence level of 95%.

**Table 3:** Coefficients and values of *R*^2^ (fitted to degrees of freedom of each fit). All with a *p*-value under 0.05.

models	*R*^2^(d.f.)	*a*	*B*	*c*	*d*	*df*	F
**M1**	99.73	8.05636·10–5	—	—	—	1	215961.39
**M2**	95.14	4.56·10–6	3.15114	—	—	1	11102.06
**M3**	97.24	7.21719·10–5	2.07092	—	—	1	2086.06
**M4**	62.40	7.57888·10–5	1.28121	—	—	1	937.82
**M5**	89.19	1.45985·10–5	2.96853	—	—	1	4662.33
**M6**	97.25	7.45313·10–5	—	—	—	1	20047.43
**M7**	97.23	3.7313·10–4	—	—	—	1	19909.76
**M8**	90.74	1.56057·10–4	0.916383	—	—	1	5555.33
**M9**	92.05	7.8085·10–6	2.97397	—	—	1	6558.30
**M10**	91.08	4.9584·10–5	1.74506	0.133815	—	2	2892.20
**M11**	95.99	1.0775·10–5	1.67757	1.26742	0.091396	3	4515.77
**M12**	92.70	7.21679·10–6	3.20805			1	7178.54
**M13**	99.00	2.512961·10–3	—	—	—	1	4857921.92
**M14**	99.98	—	0.443762			1	56262.90
**M15**	97.28	0.209187	—	—	—	1	20362.75

Validation of the models with the reserved data also involved the calculation of several indicators: the non-linear coefficient of determination fitted to degrees of freedom, mean absolute error, mean relative error and their corresponding standard errors, and lastly the mean value of the residuals. This last indicator establishes whether the estimated values adapt to the values observed or, if not, whether they under or overestimate these values (Table 4). Table 4 is completed with the results for a reference model of the relationships between tuna length and weight published recently. The model proposed in [21] is a concretion of Eq (8) and has the coefficients a = 0.0000287 and b = 2.9076 [21].

$$W=a\cdot L^{b}$$ (8)

**Table 4:** Values of the parameters defining the goodness of fit, which relate weight to tuna dimensions; calculated from the data reserved for the validation.

Model	*R*^2^(*d*.*f*.)	*Eam* (kg)	*e-Eam* (kg)	*Erm* (%)	*e-Erm* (%)	*Resm* (Kg)
Deguara et al (2016)	24.31	42.30	0.91	25.68	0.43	42.11
M1	95.17	8.77	0.36	4.57	0.21	0.16
M2	94.94	9.41	0.34	4.85	0.19	0.95
M3	94.98	9.36	0.34	4.88	0.18	1.88
M4	88.04	14.84	0.51	7.43	0.23	4.73
M5	93.19	11.47	0.37	5.99	0.19	7.08
M6	93.66	10.84	0.37	5.51	0.19	2.49
M7	93.19	10.88	0.40	5.93	0.25	7.76
M8	95.21	9.23	0.33	4.74	0.17	2.04
M9	94.58	9.11	0.39	4.70	0.22	2.36
M10	94.16	8.44	0.44	4.56	0.32	1.04
M11	95.26	8.24	0.38	4.36	0.24	0.92
M12	86.27	15.19	0.58	7.17	0.26	-13.05
M13	87.60	13.31	0.61	6.36	0.30	-4.24

**Fig 1:**
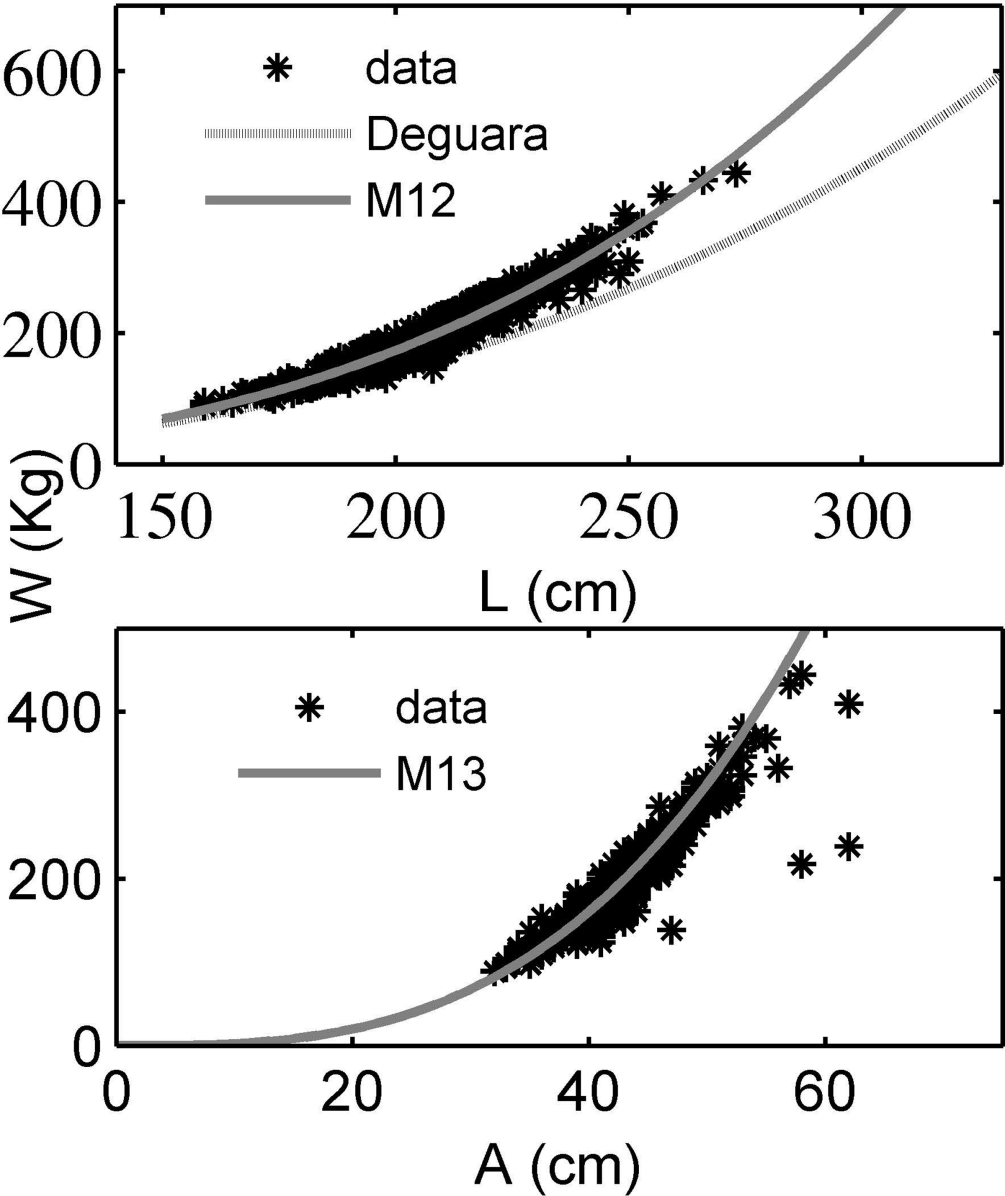
Graph of the fitted model M12, and the reference model of Deguara et al. (2016). Graph of the fitted model M13 (bottom).

Table 4 shows the goodness of fit indicators that relate weight to one or more than one dimension of tuna. Fig 1 shows the curves of all the fits considered to be a single dimension for estimating weight: the model in [21] and models M12 and M13 presented in this study.

**Fig 2:**
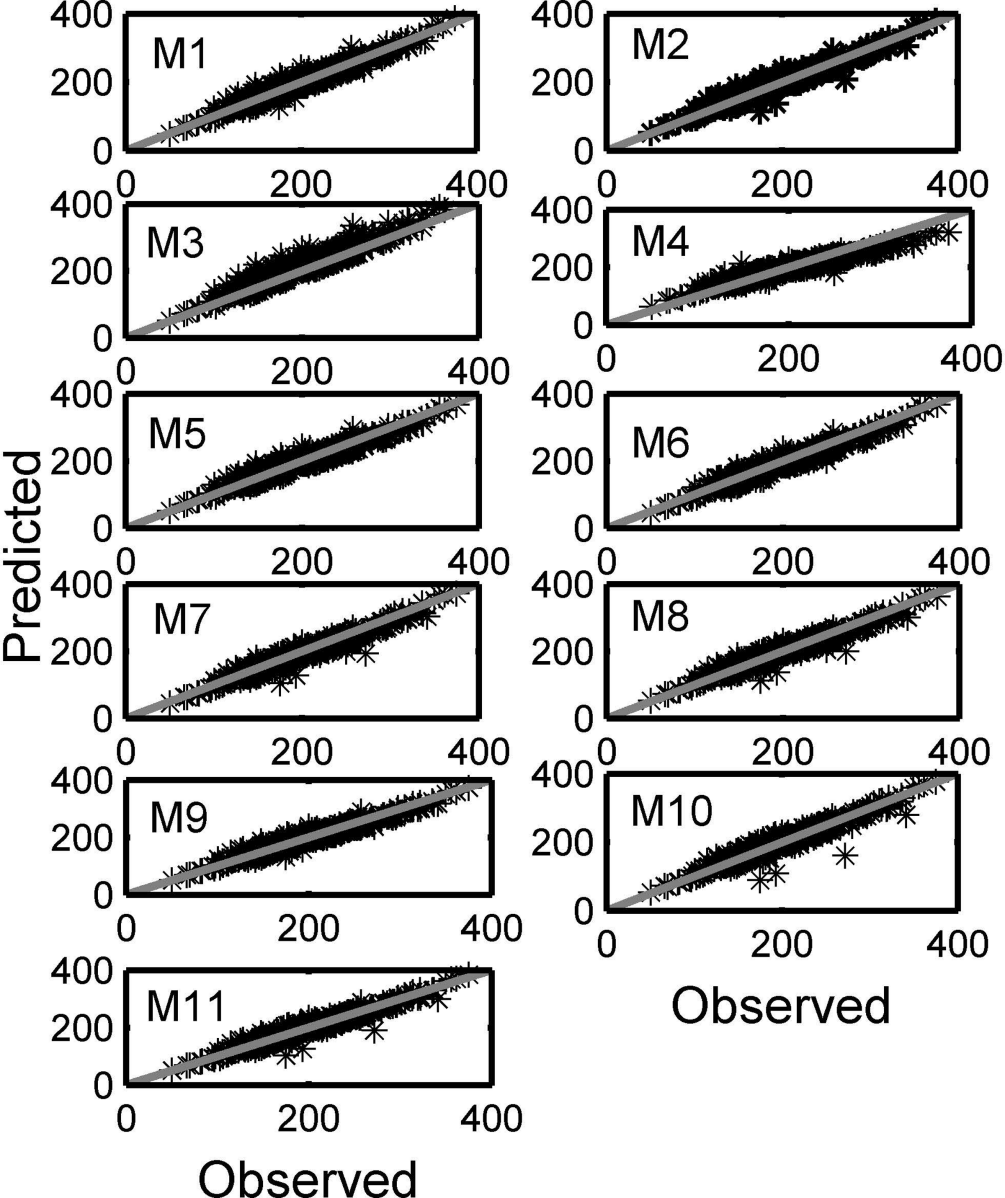
Graphs of observed weight versus predicted values for models 1 to 11.

Fig 2 shows the observed values against the predicted values with the fit made by all the models relating weight and at least two tuna dimensions (L, H and A).

The comparative analysis of models relating weight with fish dimensions was completed with the results of the ANOVA run on mean absolute error, mean relative error and mean of the residuals, corresponding to the different models analyzed (Tables 5–7). To establish the homogeneity of the groups Fisher's LSD method was used, with a 95% confidence interval.

**Table 5:** Results of applying the multiple range test to mean absolute error. The F-value corresponds to the ANOVA, which checks the equality of all the means. Also indicated is mean absolute error (in ascending order) for each model. The last column indicates the predictive variables and when a between-brackets letter coincides in consecutive two rows, it indicates that there are no statistically significant differences (95%) between the means.

*F* = 299.64, *p*<0.000 Model	Mean	Predictive Variables
**M11**	8,23629	LHA (a)
**M10**	8,43998	LHA (a)
**M1**	8,76972	LH (a)
**M8**	9,11387	LHA (a)
**M9**	9,22796	LHA (a)
**M3**	9,35717	LA (a)
**M2**	9,40722	LH (a)
**M5**	10,8446	LA (b)
**M7**	10,8837	LHA (b)
**M6**	11,466	LA (b)
**M13**	13,3114	A (c)
**M4**	14,8372	LA (d)
**M12**	15,1931	L (d)
**Deguara**	42,1138	L (e)

**Table 6:** Results of applying the multiple range test to mean relative error. F-value corresponds to the ANOVA which checks the equality of all the means. Also given is mean relative error (in ascending order) for each model. The last column shows the predictive variables and when a between-brackets letter coincides in two consecutive rows, it indicates that there are no statistically significant differences (95%) between the means.

*F* = 690.45, *p*<0.000 Models	Mean	Predictive Variables
**M11**	4,35771	LHA (a)
**M10**	4,55862	LHA (ab)
**M1**	4,56925	LH (ab)
**M8**	4,70279	LHA (ab)
**M9**	4,74283	LHA (ab)
**M2**	4,85382	LH (ab)
**M3**	4,8763	LA (ab)
**M5**	5,5126	LA (bc)
**M7**	5,9294	LHA (c)
**M6**	5,9944	LA (c)
**M12**	7,17349	L (d)
**M4**	7,42609	LA (d)
**M13**	13,3114	A (f)
**Deguara**	25,6784	A (g)

**Table 7:** Results of applying the multiple range test to the means of the residuals. F-value corresponds to the ANOVA, which checks the equality of all the means. Also indicated is the mean of the residuals for each model. The last column indicates the predictive variables and when a between-brackets letter coincides in two consecutive rows, it indicates that there are no statistically significant differences (95%) between the means.

*F* = 346.06,p<0.000 models	Mean	Predictive Variables
**M12**	-13,0472	L (a)
**M13**	-4,25704	A (b)
**M1**	0,1596	LH (c)
**M11**	0,92156	LHA (cd)
**M2**	0,95214	LH (cd)
**M10**	1,03688	LHA (cd)
**M3**	1,88382	LA (cd)
**M9**	2,03888	LHA (d)
**M8**	2,36122	LHA (d)
**M5**	2,48826	LA (d)
**M4**	4,7283	LA (e)
**M6**	7,08378	LA (f)
**M7**	7,75778	LHA (f)
**Deguara**	42,1138	L (h)

Obtaining biometric data of tuna in the wild is complicated. Nevertheless, when the fishes are held in cages for fattening the data can be obtained using different techniques. Some of them have been tested and validated for this species such as, for example, stereoscopic vision systems [5,6,7,10,16]. Table 8 summarizes the goodness of fit indicators used to validate the models relating height to length, and maximum width, and length to maximum width. Tables 4 and 8 also include the mean value of the residuals.

**Table 8:** Values of the parameters that define the goodness of fit (models 14 and 15) calculated from the data reserved for the validation.

Model	*R*^2^(*d*.*f*.)	*Eam* (kg)	*e-Eam* (kg)	*Erm* (%)	*e*^*Er*^(%)	*Resm* (Kg)
**M14**	86.06	1.55	0.07	2.73	0.12	0.21
**M15**	84.94	1.24	0.05	2.89	0.11	-0.40

**Fig 3:**
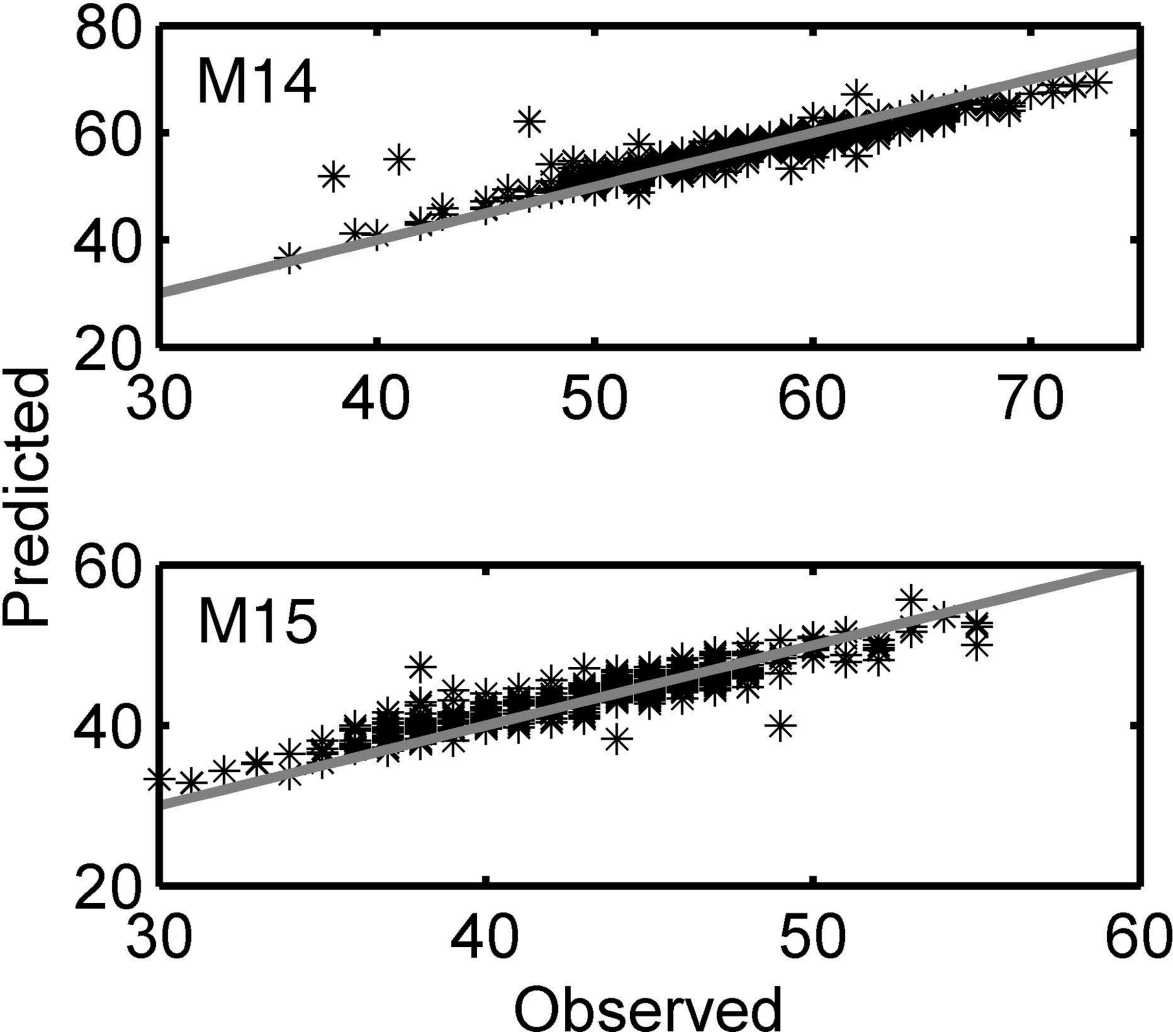
Graphs of observed values against those predicted for models M14 and M15. M14 provides height (*H*) predicted values in relation to observed values, M15 offers maximum width (*A*) predicted values in relation to observed values.

To complete the representation of the results, Fig 3 shows the graphs of observed values versus predicted values for models M14 and M15.

## Discussion

Table 4 shows the values of the goodness of fit indicators that relate weight with one or more than one dimension of the tuna. The results indicate that the availability of fattened tuna height or width improves the predictive power of the models, given that the values of the coefficient of determination increase at the same time as both mean absolute error and mean relative error decrease. The same thing happens if we consider the three dimensions (length, height and width). In the case of considering maximum width and length, coefficient of determination values are slightly lower but so are the corresponding mean absolute error and mean relative error values in relation to those obtained when just one dimension is used in the model. Model 13 (M13) provides a good fit between tuna weight and maximum width. Reviewing the goodness of fit values shows low absolute errors and low relative errors, comparable with those obtained when considering length as the only dimension in the fit.

If we look at Table 5, showing the ANOVA analysis on mean absolute error values for all the fitted models and those of reference [21], it can be seen that the introduction of more than one dimension in the fit reduces the mean absolute error value. Clearly the introduction of three dimensions provides the lowest absolute errors (as is the case of M10 and M11). The same table shows how models M11, M10, M1, M8, M9, M3 and M2 provide absolute errors lower than 10 Kg. If we look at Table.6, which presents the values of the ANOVA analysis on the relative errors, we can see that these same models (M11, M10, M1, M8, M9, M3 and M2) give weight estimates with errors of less than 5%, acceptable values in the same range as those offered by indirect systems of length estimation such as those involving stereoscopic cameras [5,6,7]. Furthermore, both in the Tables 5 and 6 is confirmed that using a single dimension increases error in the prediction of weight, though it should be pointed out that using the maximum width instead of length does not significantly increase the error, as revealed by the mean absolute and relative error values of M12, M13 and the reference model of [21]. Nevertheless, it is very important to remember that the reference model [21] was obtained for the weight of wild fishes, usually before or during the spawn, rather than fishes later caught and fattened in captivity. In addition, it is more than plausible to assume that, in the case of wild fishes, length is the most determinant dimension for predicting weight. Furthermore, in fishes fattened in captivity, dimensions are affected by life conditions, such that the remaining dimensions, in particular width, gain relevance as predictive variables of weight.

In the same way, Table 7 reflects the result of the ANOVA analysis of the mean value of the residuals. In this case as in the previous ones, the smallest deviations occur in the fits M1, M11, M2, M10, M3, M9, M2 and M5, all of which have values of less than 3, with values exceptionally low and less than 1 for fits M1, M11 and M2. This confirms that increasing the number of dimensions in the weight determination improves the predictions offered by the models. Furthermore, the more usual expressions for the estimates of the weight of fishes in the wild require an adaptation to fit the values obtained with fishes fattened in captivity, since they tend to overestimate weight. On the other hand, when the data are fitted to a single dimension (length, L, or width, A) as M12 and M13 show, mean weight predictiontend to underestimate mean weight in predictions.

As already indicated, from the analysis of the mean values of the residuals it is inferred that most of the models overestimate the weight of the tuna, however, this overestimation diminishes when more than one dimension of tuna is included, and is close to zero for M1, which only includes height. Models M12 and M13 underestimate weight. M12 only uses length as an independent variable and M13 uses only width. The fact that the mean value of the residuals is close to zero indicates the reliability of the prediction offered by the fit. But in the case of the determination of height from length and width (M14), the mean value of the residuals is practically zero, which, together with error values of less than 3%, points to the possibility of obtaining this dimension indirectly from the other two. In the same way model 15 indicates that there is a relationship between the length and width of fattened fishes, which is confirmed by the low errors in the prediction and residuals close to zero.

In Fig 1, which shows the models fitted with just one dimension, a review of the expressions used for wild fishes is needed in order to adapt them to fishes fattened in cages. In this same graph and in Table 4 we can see that the use of width as the only variable to predict weight offers good results, with errors of less than 10%.

## Conclusions

Regression models have commonly been used to determine the weights of tuna from their lengths. This study has demonstrated that the use of more than one dimension improves weight estimation when fish have been fattened over months in captivity. The use of height as an additional dimension is more common when working with biometrics, mainly using stereoscopic vision systems but, the ANOVA analysis in this work shows that the maximum width of tuna can be used as a parameter for the determination of weight together with length. The use of maximum width offers good coefficients of determination and mean error values similar to those that can be achieved using height, as shown by the comparison of the presented models (M1 and M3).

When the three dimensions of the fish are used (length, height and maximum width), goodness of fit improves, reducing absolute error to below 9 kg and obtaining relative errors of less than 5%. The values of the mean residuals of less than 1 indicate the high degree of prediction obtained by using the three dimensions of tuna to estimate its weight. These results seem to indicate that measurements of the three dimensions should be taken in recently captured fishes in order to improve estimation of their state according to geographical location and season of the year.

In addition, a model that permits the indirect determination of height from length and width is presented that can be applied in new counting and sizing techniques during the ventral recording of images of tuna during transfers.

Similarly, models are obtained that enable the relationship between fish length and maximum width to be established. The results in this case present a clear relationship between width and length, such that mean absolute errors of 1.25 cm are obtained when estimating width from length, which indicates prediction errors of less than 3%. Such small errors, together with the mean values of the residuals (close to zero), indicate a strong relationship between the two dimensions that can be used to eliminate outliers from future studies.

To conclude, we suggest the extension of this study to different tuna and commercial species in order to improve the quality of catch estimates and production management tools in aquaculture.
