# Correction: Relationship between weight and linear dimensions of Bluefin tuna (*Thunnus thynnus*) following fattening on western Mediterranean farms

**DOI:** 10.1371/journal.pone.0259437

**Published:** 2021-10-27

**Authors:** Vicente Puig-Pons, Vicente Domingo Estruch, Víctor Espinosa, Fernando de la Gándara, Begonya Melich, José Luis Cort

In the third column of Table 3, the exponents listed in the fifth, ninth, eleventh, and twelfth row are incorrect. Please see the correct Table 3 here.

**Table 3:** Coefficients and values of *R*^2^ (fitted to degrees of freedom of each of the fits). All with a *p*-value under 0.05.

models	*R*^2^(d.f.)	*a *	*B *	*c *	*d *	*df *	F
M1	99.73	8.05636·10–5	—	—	—	1	215961.39
M2	95.14	4.56·10–6	3.15114	—	—	1	11102.06
M3	97.24	7.21719·10–5	2.07092	—	—	1	2086.06
M4	62.40	7.57888·10–3	1.28121	—	—	1	937.82
M5	89.19	1.45985·10–5	2.96853	—	—	1	4662.33
M6	97.25	7.45313·10–5	—	—	—	1	20047.43
M7	97.23	3.7313·10–4	—	—	—	1	19909.76
M8	90.74	1.56057·10–3	0.916383	—	—	1	5555.33
M9	92.05	7.8085·10–6	2.97397	—	—	1	6558.30
M10	91.08	4.9584·10–4	1.74506	0.133815	—	2	2892.20
M11	95.99	1.0775·10–4	1.67757	1.26742	0.091396	3	4515.77
M12	92.70	7.21679·10–6	3.20805			1	7178.54
M13	99.00	2.512961·10–3	—	—	—	1	4857921.92
M14	99.98	—	0.443762			1	56262.90
M15	97.28	0.209187	—	—	—	1	20362.75
